# Fingerprint Feature Extraction for Indoor Localization †

**DOI:** 10.3390/s21165434

**Published:** 2021-08-12

**Authors:** Jehn-Ruey Jiang, Hanas Subakti, Hui-Sung Liang

**Affiliations:** Department of Computer Science and Information Engineering, National Central University, Taoyuan City 320317, Taiwan; hanas@g.ncu.edu.tw (H.S.); ky830528@gmail.com (H.-S.L.)

**Keywords:** autoencoder, Bluetooth, beacon, fingerprint indoor localization, principal component analysis

## Abstract

This paper proposes a fingerprint-based indoor localization method, named FPFE (fingerprint feature extraction), to locate a target device (TD) whose location is unknown. Bluetooth low energy (BLE) beacon nodes (BNs) are deployed in the localization area to emit beacon packets periodically. The received signal strength indication (RSSI) values of beacon packets sent by various BNs are measured at different reference points (RPs) and saved as RPs’ fingerprints in a database. For the purpose of localization, the TD also obtains its fingerprint by measuring the beacon packet RSSI values for various BNs. FPFE then applies either the autoencoder (AE) or principal component analysis (PCA) to extract fingerprint features. It then measures the similarity between the features of PRs and the TD with the Minkowski distance. Afterwards, *k* RPs associated with the *k* smallest Minkowski distances are selected to estimate the TD’s location. Experiments are conducted to evaluate the localization error of FPFE. The experimental results show that FPFE achieves an average error of 0.68 m, which is better than those of other related BLE fingerprint-based indoor localization methods.

## 1. Introduction

Indoor localization is a procedure of locating or positioning a target device (TD) in indoor environments, such as buildings, houses, stores, and factories. It has become an important aspect in wide-scale applications including the health, industry, commerce, surveillance, and various sectors [1]. For example, in the health sector, indoor localization can help the elderly, the handicapped and the visually impaired to navigate inside the hospital [2]. In another example, indoor localization can be used for assisting living applications like behavioral monitoring and fall detection for elderly people and disabilities [3]. For yet another example, indoor localization can also contribute to the industry, such as robot navigation, asset tracking, and workpiece location monitoring for production control [4].

Indoor localization could be developed by using various categories of technologies such as optics, infrared (IR), mechanical sensor (MS), and radio frequency (RF) technologies [5,6]. An optical or vision-based localization system takes advantage of a TD’s camera and computing capacity. The interference from numerous factors such as strong light, motion blur, and larger accumulative mistakes all contribute to the system’s poor performance [6]. The IR technology was used at early indoor localization systems [7]. The line-of-sight (LOS) restriction and limited device compatibility are significant drawbacks of IR-based systems [8]. TD’s built-in mechanical sensors like the accelerometer, magnetometer, and gyroscope can also be utilized to realize indoor localization. The fluctuations and errors accumulated during the sensors capture data can degrade the localization accuracy especially in large location areas [9]. RF-based localization systems use RF signals to determine TD’s location [10]. Some types of RF signals can penetrate walls and obstacles, so RF-based localization systems can have large coverage areas. Furthermore, many TDs incorporate RF technologies by default, resulting in relatively low costs. Because of all the above-mentioned advantages, this research focuses on localization methods using RF technologies.

Several RF technologies, such as Bluetooth low energy (BLE) [11], ultra-wideband (UWB) [12], Wi-Fi [13], and cellular [14], have been used in indoor localization. Among these technologies, the BLE technology is designed for short-range wireless communication with low energy consumption, low cost, and easy deployment. Although UWB devices consume very low energy as BLE devices do, UWB is not as widely supported. Wi-Fi is as widely supported as BLE and has larger coverage than BLE and UWB, but it has much more energy consumption than BLE. Cellular technology has a much larger coverage than others, but it consumes more energy and requires vast investment in the infrastructure of extensive base stations. This research thus focuses on applying the BLE technology to develop indoor localization methods. Many BLE indoor localization methods [15,16,17,18,19,20,21,22,23,24,25,26,27,28,29,30,31,32,33] have been proposed in the literature. Among them, BLE fingerprint-based methods have comparably good localization accuracy. Hence, this research aims at developing BLE fingerprint-based indoor localization methods.

For the purpose of locating a target device (TD), whose location is unknown, fingerprint-based indoor localization methods deploy at proper locations some beacon nodes (BNs) that periodically broadcast beacon packets containing the BN’s ID, location, and other information [34]. Devices receiving beacon packets can easily obtain the received signal strength indication (RSSI) values and other information of the packets. Fingerprint-based indoor localization methods usually consist of two main phases: offline and online. The offline phase is to collect beacon packet RSSI values from different BNs for each reference point (RP) with a known location. These collected RSSI values are fingerprints of RPs. They, along with corresponding RP locations, are stored in the database as the reference fingerprinting map (RFM). The online phase is to collect beacon packet RSSI values of different BNs as a TD’s fingerprint and compare it with those stored in the database. The RPs having the most similar fingerprints with the TD’s are identified with a matching algorithm. Then, the TD’s location is estimated based on the locations of the identified RPs.

Five BLE fingerprint-based indoor localization methods [16,22,23,24,27] that are most related to our research are introduced below. Zuo et al. [16] adopted graph optimization to perform indoor localization and produced an error of 1.27 m in the best case. Martins et al. [22] performed indoor localization using Gaussian kernel-based fingerprinting and achieved errors that are less than 1.5 m for approximately 90% of test cases. Subedi et al. [23] employed an improved two-step fingerprint-based localization method, resulting in a localization error of 1.05 m. Li et al. [24] predicted the TD location with an eight-neighborhood template-matching mechanism and achieved a localization error of 1.0 m. Dinh et al. [27] proposed a lightweight and reliable fingerprint-based method using pedestrian dead reckoning and trilateration, bringing about an average error of 0.81 m.

This paper proposes a fingerprint-based indoor localization method, named fingerprint feature extraction (FPFE), using the BLE technology. Four BLE BNs are deployed in an indoor environment to emit beacon packets periodically. The beacon packet RSSI values of the four BLE BNs are measured at different RPs and stored in the fingerprint database. RSSI measurements have the problem that they are susceptible to interference, multipath, signal noise, and so on. Feature extraction [35] can mitigate the RSSI measurement problem by extracting representative features from RSSI measurements. It can also accelerate fingerprint matching by using only a few features, instead of a lot of RSSI measurements, in fingerprint matching. The FPFE method first uses either the autoencoder (AE) [36] or principal component analysis (PCA) [37] for feature extraction of beacon fingerprints. It then calculates the Minkowski distances [38] between the feature of a TD and the features of all RPs. Afterwards, *k* RPs associated with the smallest Minkowski distances are selected and their centroid is assumed to be the TD’s location. Experiments are conducted to evaluate FPFE’s performance. FPFE is also compared with the most related methods [16,22,23,24,27] to show its superiority. Furthermore, a practical application using FPFE for smart living is also introduced to show the applicability of FPFE.

The contributions of this paper are fourfold. First, it proposes the FPFE method utilizing BLE, an RF technology that is inexpensive and energy-efficient and can be run on smart mobile devices without costly deployment of wiring. Second, the proposed method achieves submeter localization errors by using AE, PCA, and the Minkowski distance to perform fingerprint-based indoor localization. Third, extensive experiments have been conducted to show how the performance of FPFE is influenced by the setting of RPs. Fourth, the proposed method is employed to realize a practical application to show its applicability.

Note that this paper is an extended version of the research article [39]. This paper has the following extensions. First, it demonstrates extensive experimental results of FPFE for different RP settings. Second, an application using FPFE is introduced in this paper. Third, this paper contains thorough discussions of FPFE and numerous research directions of indoor localization using advanced technologies.

The rest of this paper is organized as follows. Section 2 introduces five fingerprint-based indoor localization methods [16,22,23,24,27] using the BLE technology. The proposed FPFE method is elaborated in Section 3. Section 4 describes experiment details of performance evaluation and comparisons. Finally, the paper is concluded in Section 5.

## 2. Related Work

Zuo et al. [16] proposed an indoor localization method adopting the graph optimization concept. The method is fingerprint-based; it is also range-based, meaning that it relies on RSSI values to estimate the ranges (distances) between the TD and BNs. A TD moving around a region collects inertial measurements and RSSI readings. Constraints of adjacent TD’s poses (i.e., positions) are generated by processing the inertial measurements with the pedestrian dead reckoning (PDR) mechanism. The RSSI readings are used as ID’s fingerprints to generate other constraints of adjacent TD’s poses. The readings are also used to generate distance constraints between BN locations and the TD’s poses. The constraints are altogether adopted to form a cost function of a least-square form. The TD’s poses at different times, the reference fingerprint map, and BN locations can be optimally estimated by graph optimization. Specifically, graph optimization is the process of minimizing the cost function and representing the relationships between variables associated with the inertial and RSSI readings. Experiments are performed in an area of 90 m × 37 m with different numbers of BNs, 24 BNs in a sparse mode, and 48 BNs in the dense mode. The experimental results show that the accuracy of the errors are 2.26 and 1.27 m in the sparse and the dense beacon environments, respectively.

Martins et al. [22] proposed a localization method using BLE RSSI fingerprints. The proposed method was carried out on the Viseu Polytechnic Institute campus with hundreds of students (users), each with a smartphone (i.e., TD) to navigate in a building. The method relies on a database storing all beacons identifiers and wall (obstacle) conditions. The TD uses received signals of BNs to search for matched BNs stored in the database. The TD’s location is assumed to be unknown if there is no matched signal of known BNs. However, if only one matched BN is found, then the distance between the TD and the BN is calculated. If the distance is less than a threshold (specifically 2 m), then the method returns the estimated position relative to the BN. On the other end, if the distance is larger than the threshold, then the TD’s position is also assumed to be unknown. Suppose there are two matched BNs, two probable locations are calculated, which correspond to the intersections of two circles cantered on the two BNs, either with an estimated distance. The method checks if the wall conditions hold. If so, the BN with the stronger RSSI is chosen to determine the TD’s location. Furthermore, if there are three or more matched BNs, then the BNs with the top three signal strength are chosen to determine the TD’s location with the trilateration mechanism. Finally, by combining the Bayesian estimator and the Gaussian kernel model and the concept of fingerprinting, the method determines TD’s location during a user walk. Experimental results of the proposed method show that it achieves errors that are less than 1.5 m for approximately 90% of test cases in a 200 m × 40 m testbed floor plan.

To increase the indoor localization accuracy, Subedi et al. [23] presented an improved two-step fingerprinting localization method using multiple fingerprint features. This method transforms BLE RSSI to distance according to the propagation model and then estimates the weighted centroid (WC) of nearby BNs. Instead of RSSI from all BNs, the estimated WCs, signal strength, and rank of the nearby BNs are saved in the server database for the purpose of localization. This method employs a variety of fingerprinting features to improve localization accuracy and reduce the physical size of the database and the amount of data communication. The method also utilizes affinity propagation clustering to reduce the searching space of RPs and decrease computational costs. Furthermore, exponential averaging is used for smoothing the noisy RSSI. Experimental results show that the method can significantly reduce the radio-map database size and improve the positioning accuracy with the best localization error of 1.05 m.

Li et al. [24] proposed an algorithm based on eight-neighborhood template matching to solve BLE signal non-line-of-sight propagation and other issues that affect indoor localization accuracy. The algorithm first divides the indoor environment into four quadrants for each BN, called an access point (AP) in [12]. Then, the expected values of the RSSI difference between the centre points and their eight-neighborhoods are calculated. The values are used to calculate templates for RPs and the TD. Finally, template matching is applied to choose the best RP whose template is most similar to that of the TD for the estimation of TD location. By experiments performed in an 8 m × 8 m room, the method can achieve a 1.0 m localization error.

Dinh et al. [27] proposed an indoor localization method using trilateration, pedestrian dead reckoning (PDR), and fingerprinting. The trilateration and PDR mechanisms are used to estimate the TD initial location and current location, respectively. The fingerprinting mechanism is based on a lightweight and reliable fingerprint map to correct TD initial location estimation errors and orbital drifts. The map is lightweight because the mechanism produces the map by collecting data from only a small number of RPs, instead of dividing the map into high-resolution grids with a huge number of RPs, therefore significantly reducing the amount of time to deploy the system. The map is also reliable, it produces good precision by using feature vectors and a matching algorithm to find three nearest RPs based on each RP’s RSSI profile defined in the map. Finally, these three RPs coordinates are combined and calculated through a particle filter to correct the PDR error. Experiments were conducted in a 15 m × 25 m area to show the proposed method achieves an average error of 0.81 m.

## 3. Proposed Method

**Figure 1:**
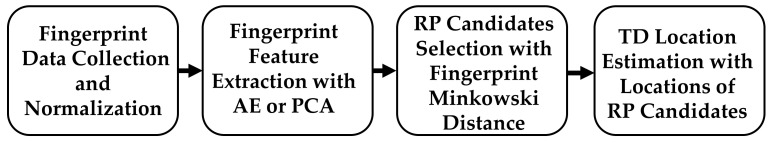
The major steps of the proposed FPFE localization method.

The major steps of the proposed FPFE localization method are depicted in Figure 1. The method starts with collecting RPs BLE beacon RSSI values to build a fingerprint database. Note that RPs are assumed to be distributed over the whole localization area with an irregular pattern (e.g., a random pattern) or with a regular pattern (e.g., a grid pattern). Then, either the AE or PCA is applied for fingerprint feature extraction. The Minkowski distance is used as the fingerprint similarity measurement to select *k* RP candidates with the smallest distances. The TD location is then calculated by averaging coordinates of the *k* selected RP candidates. Each major step is described in a separate section below.

### 3.1. Fingerprint Data Collection and Normalization

**Figure 2:**
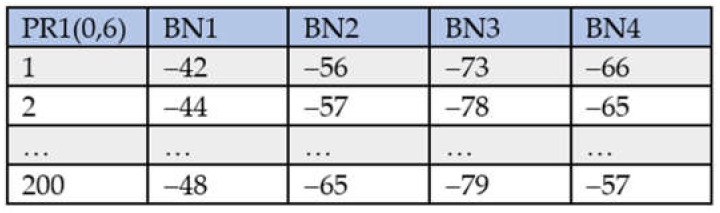
BLE RSSI fingerprint data for a reference point. Adapted from ref. [39]. © IEEE 2021.

For each RP, 200 fingerprint data are collected. For example, Figure 2 shows the data for reference point 1 (RP1), whose location is (0,6). In the figure, each row represents one fingerprint containing four RSSI values for four BNs, BN1, BN2, BN3, and BN4. Bluetooth enabled smartphones are used to measure the RSSI values which range from $x_{max}$ (−20) to $x_{min}$ (−100). A smaller value indicates a weaker BN signal received. The min-max scaling method is applied to the data for the purpose of normalization. The mathematical formulation of min-max scaling is as follows:

(1) $$x_{norm}=\left(x-x_{min}\right)/\left(x_{max}-x_{min}\right),$$

where $x_{norm}$ ranging from 0 to 1 is the normalization value, *x* is the original RSSI value, and $x_{min}$ and $x_{max}$ are the minimum and maximum of RSSI values, respectively.

### 3.2. Fingerprint Feature Extraction with AE or PCA

Feature extraction is a process of dimensionality reduction. It can project an initial set of data in high-dimension space to be data in low-dimension space without losing critical information. It is useful for efficiently processing large datasets that require a lot of computing resources.

The proposed method uses either the AE or PCA for feature extraction of beacon fingerprint data. Note that fingerprint data for an RP are of the shape of 4 × 200. They are transformed to be of the shape of 1 × 800 as the input of the AE or PCA.

#### 3.2.1. AE Feature Extraction

**Figure 3:**
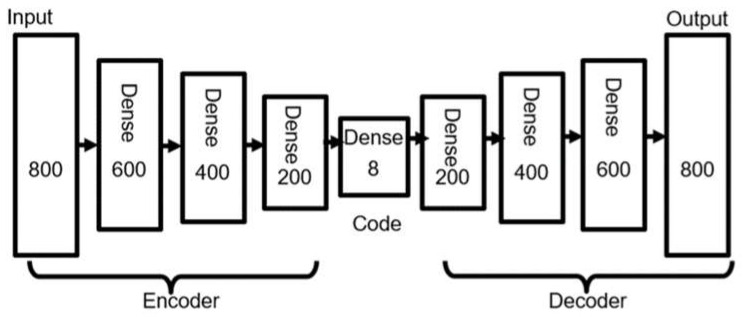
The autoencoder structure adopted from the FPFE method.

An AE is a special artificial neural network (ANN) model that encodes higher-dimension input features to be a lower-dimension internal representation called the code. An AE model consists of three parts: the encoder, the code, and the decoder. The encoder processes the input features to generate the code, and the decoder then processes the code to generate the reconstructed input features as the output. Figure 3 is the AE structure adopted by the proposed FEFE method. The encoder in the AE in Figure 3 takes 800 features as the input and has three dense (i.e., fully connected) neural layers of 600, 400, and 200 neurons, respectively. The code is a dense layer of 8 neurons. The decoder has three dense layers of 200, 400, and 600 neurons, respectively, and generates 800 features as the output.

Generally, the encoder and the decoder of an AE have several neural layers and have symmetric structures, as exemplified by the AE in Figure 3. For the sake of simplicity, we explain below the process of an AE whose encoder and decoder have only one neural layer. When an input vector *x* is fed into the AE, it is transformed into a vector *z* as the code by the first half part (i.e., the encoder part) of the neural network. Then, from the code vector *z*, the last half part (i.e., the decoder part) of the neural network tries to reconstruct *x* as a vector *x’*. Given the input vector *x* ∈$\;{\mathbb{R}}^{d}$ and the encoded vector *z* ∈ ${\mathbb{R}}^{d^{\prime }}$, the encoding and decoding processes of the AE are mathematically formulated as follows:

(2) $$z=\sigma \left(W_{encoder}\;x+b_{encoder}\right),$$

where σ is a nonlinear activation function, e.g., sigmoid, hyperbolic tangent, or rectified linear unit (ReLU), and $W_{encoder}\in {\mathbb{R}}^{d^{\prime }\times d}\;\mathrm{and}\;b_{encoder}\in {\mathbb{R}}^{d^{\prime }}$ are respectively the weights and the bias of the single neural layer of the encoder. The output $x^{\prime }\in {\mathbb{R}}^{d}\;$of the AE is formulated as follows:

(3) $$x^{\prime }=\sigma \left(W_{decoder}\;z+b_{decoder}\right),$$

where σ is a nonlinear activation function, and $W_{decoder}\in {\mathbb{R}}^{d\times d^{\prime }}\;\mathrm{and}\;b_{encoder}\in {\mathbb{R}}^{d}$ are respectively the weights and the bias of the single neural layer of the decoder. The weights of the AE are restricted by setting $W_{decoder}=W_{encoder}^{t}$, where $W_{encoder}^{t}$ is the transpose of $W_{encoder}^{}$, so the number of neural network weights is reduced by half. In general, an AE model has the encoder and decoder, each with multiple symmetrical neural layers of restricted weights. The difference between the input *x* and the output *x’* (i.e., the reconstruction of *x*) is regarded as the reconstruction error. Like other ANNs, the AE model updates weights of the AE model by minimizing the reconstruction error with the backpropagation algorithm. The AE model is ready for use after minimizing the reconstruction error between the input and the output. The low-dimensional code can then be used as a good extraction of the high-dimensional input of features.

As shown in Figure 3, the BLE BN RSSI values measured at an RP are used as the input with the dimension of 800 × 1 in this research. The input is encoded as a code with the dimension of 8 × 1, which in turn is decoded as the output with the dimension of 800 × 1. More specifically, the AE model uses the hyperbolic tangent (tanh) function as the activation function, uses the adaptive moment estimation (Adam) as the optimizer, and uses the mean squared error (MSE) as the loss function of the reconstruction error.

#### 3.2.2. PCA Feature Extraction

PCA is a dimensionality reduction and feature extraction method to project data in a higher dimensional feature space to be data in a lower dimensional feature space without losing critical information. The basic concept of a PCA is to find the first, the second, …, and the *c*th principal components that are orthogonal vectors on which data are projected for achieving the largest variance, the second largest variance, …, and the *c*th largest variance. PCA can be realized as follows. Given a set $X=\{x_{1},x_{2},\ldots ,\;x_{n}$} of *n* original data in the *d*-dimension feature space, PCA is to find a *d* × *c* transformation matrix (or projection matrix) *W* to project data into the *c*-dimension feature space such that projected data have maximal variance totally, where *c* << *d*. The data mean and standard deviation for each feature are derived to standardize the original data. The standardized data are then used to derive a *d* × *d* covariance matrix. Afterwards, *d* eigenvectors of the covariance matrix are derived, where each eigenvector is associated with an eigenvalue. The eigenvectors are sorted according to the descending order of their eigenvalues. Afterwards, the first *c* eigenvectors$\;v_{1},v_{2},\ldots ,\;v_{c}\;$with eigenvalues $e_{1},e_{2},\ldots ,\;e_{c}$ are selected as *c* principal components to be combined to construct the projection matrix *W*. Note that the eigenvalue *e_i_*, 1 ≤ *i* ≤ *c*, associated with eigenvector *v_i_* is actually the variance associated with *v_i_* when data are projected onto *v_i_*. According to Equation (4), the value of *ρ* is derived, where *ρ* is the ratio of the summation of the *c* eigenvalues over the summation of the *d* eigenvalues. The ratio *ρ* is called the explained variance ratio or the cumulative proportion. The explained variance ratio *ρ* should exceed a specific threshold *θ* (e.g., 0.9 or 0.95) so that the *c* principal components associated with the *c* eigenvectors can explain (or represent) the total data variance well enough.

(4) $$\rho =\frac{\sum_{i=1}^{c}\;e_{i}}{\sum_{i=1}^{d}\;e_{i}}$$

Finally, each original *d*-dimensional data sample $x_{i}$ can be projected onto the *c* principal components to be a new *c*-dimensional data sample $x_{i}^{\prime }$ according to Equation (5). It can be seen that the original *d*-dimensional data sample $x_{i}$ has *d* data features and the new *c*-dimensional data sample $x_{i}^{\prime }$ has *c* data features. In this way, the purpose of data dimensionality reduction and data feature extraction is achieved.

(5) $${\left(x_{i}^{\prime }\right)}_{1\times c}={\left(x_{i}\right)}_{1\times d}\;{\left(W\right)}_{d\times c},\;1\leq i\leq n$$

### 3.3. RP Candidates Selection with Fingerprint Minkowski Distance

In this study, the Minkowski distance [38] is applied to calculate similarity between features of a RP and features of a TD. Generally, the Minkowski distance is a similarity measurement between two points in a normed vector space. Let *x* = ($x_{1},x_{2},\ldots \;,x_{c})\;$and *y* = ($y_{1},y_{2},\ldots \;y_{c})$ be two points in a normed *c*-dimensional space. The Minkowski distance $D\left(x,y\right)$ between *x* and *y* is defined by Equation (6).

(6) $$D\left(x,y\right)={\left(\sum_{i=1}^{c}{\left|x_{i}-y_{i}\right|}^{p}\right)}^{1/p}$$

The Minkowski distance is also known as the L*p* norm distance. When *p* = 1, it becomes L_1_ norm, also known as the Manhattan distance. When *p* = 2, it becomes L_2_ norm, or called the Euclidean distance.

In the FPFE methods using AE feature extraction, the *p*-value is 8, because the feature extraction output of the AE has 8 features. When the PCA feature extraction is applied, the *p*-value is 7, since the feature extraction output of PCA has 7 features. The *p*-value does not need to match with the number of futures. Coincidently, taking the *p*-value as the number of features has good performance in the FPFE method.

By calculating the Minkowski distance between features of the TD and all RPs, *k* RPs with *k* smallest Minkowski distances are selected. They are called RP candidates whose locations are used to estimate the TD’s location, which will be described in the next subsection.

### 3.4. TD Location Estimation with Locations of RP Candidates

The last step of the proposed FPFE method is to estimate the TD’s location based on the locations of *k* RP candidates. Let $\left(x_{i},y_{i}\right),\;1\leq i\leq k,$ denote the location of *i*th RP candidate. The TD’s location (*x*, *y*) is calculated simply as the centroid of the *k* RP candidates, as shown in Equation (7). Different *k* values lead to different location estimations, as will be shown later.

(7) $$\left(x,y\right)=\frac{1}{k}\overset{k}{\underset{i=1}{\sum }}(x_{i},y_{i})$$

## 4. Experiments, Performance Evaluation, and an Application

### 4.1. Experimental Settings

**Figure 4:**
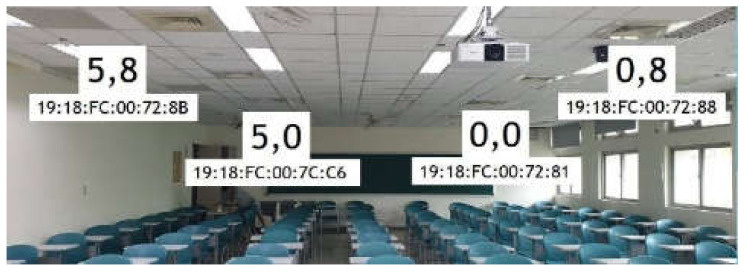
The experimental environment for the FPFE method in a 5 m × 8 m area. Reprinted from ref. [39]. © IEEE 2021.

**Figure 5:**
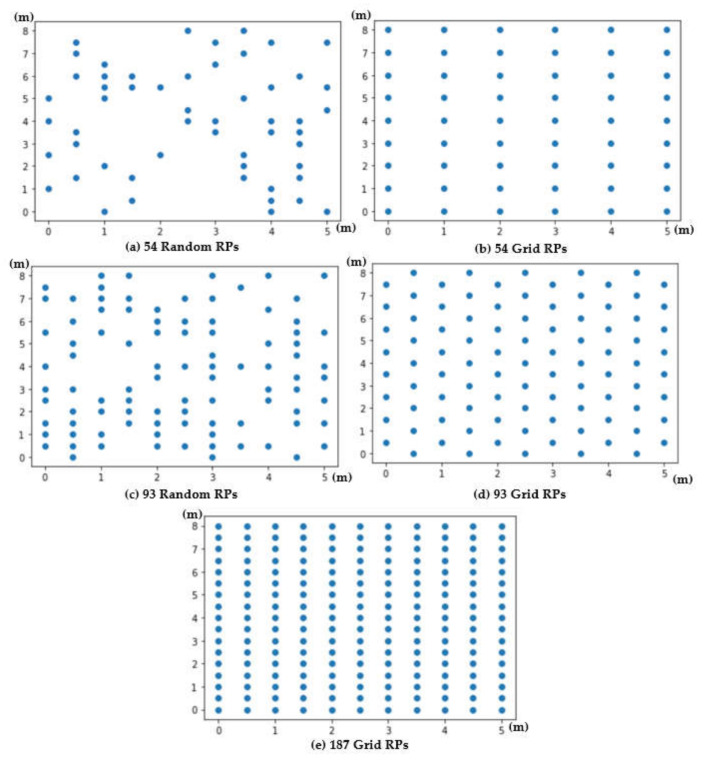
Different experimental scenarios of (**a**) 54 random RPs, (**b**) 54 grid RPs, (**c**) 93 random RPs, (**d**) 93 grid RPs, and (**e**) 187 grid RPs.

**Figure 6:**
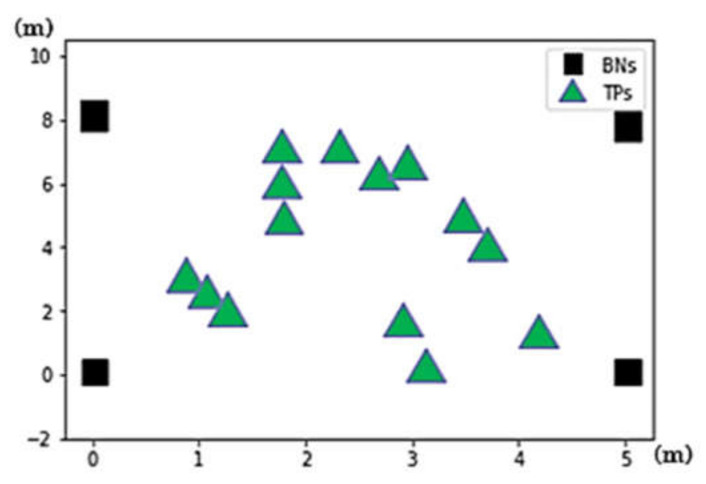
The Illustration of 12 test points (TPs) and 4 beacon nodes (BNs).

Experiments are conducted in the A303 classroom of Engineering Building V of National Central University. Four BLE BNs with coordinates (0,0), (0,8), (5,0), and (5,8) are deployed at four corners on the ceiling in a 5 m × 8 m area, as shown in Figure 4. RPs are specified right beneath the area. Five experimental scenarios with different RP settings are adopted to conduct experiments. The scenarios are 54 random RPs, 54 grid RPs, 93 random RPs, 93 grid RPs, and 187 grid RPs, as shown in Figure 5. Furthermore, 12 arbitrary locations are set as test points for evaluating FPFE performance (i.e., the localization error), as shown in Figure 6. An Android application has been developed to measure RSSI values of beacon packets sent by the BLE BNs. The Asus ZenFone 2 Laser smartphone (Asus, Taipei, Taiwan) is used to run the application. The height of the phone is set as 1 m above the floor since users in the room usually place their phones on the top of desks with about 1 m height.

**Figure 7:**
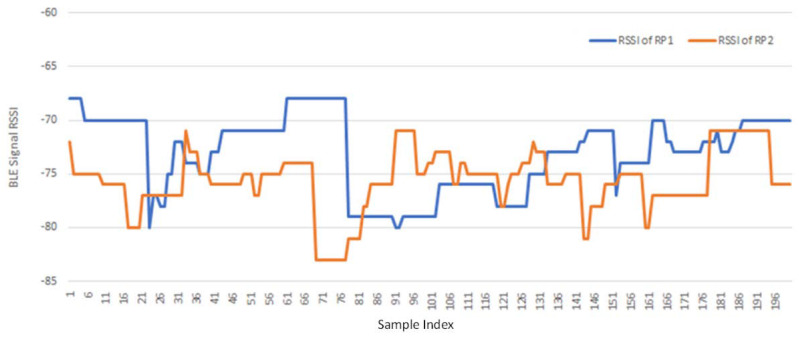
Partial fingerprint data of 200 samples recorded at two RPs for a BN.

Figure 7 shows an example of partial RSSI fingerprint data (signals) of 200 samples recorded at two RPs, RP1 and RP2, for a specific BLE BN. It can be observed that the signals are not stable. They fluctuate between −65 dB and −85 dB. This may be due to the interference coming from surrounding environments. The instability of fingerprint data in practice raises big challenges to fingerprint-based indoor localization methods.

### 4.2. Performance Evaluation

**Figure 8:**
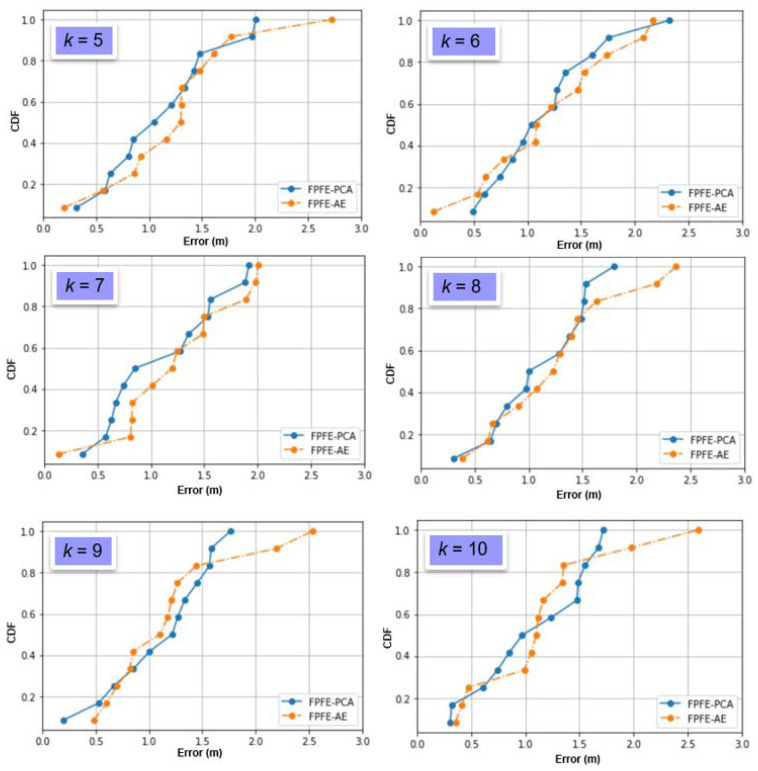
The localization error CDF curves of FPFE with 54 random RPs for *k* RP candidates, where *k* = 5, 6, 7, 8, 9, and 10.

**Figure 9:**
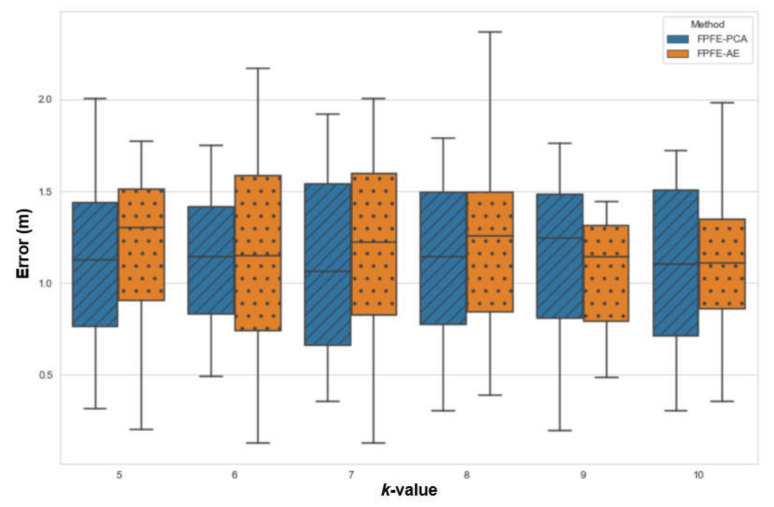
The localization error box-whisker plots of FPFE with 54 random RPs for *k* RP candidates, where *k* = 5, 6, 7, 8, 9, and 10.

**Figure 10:**
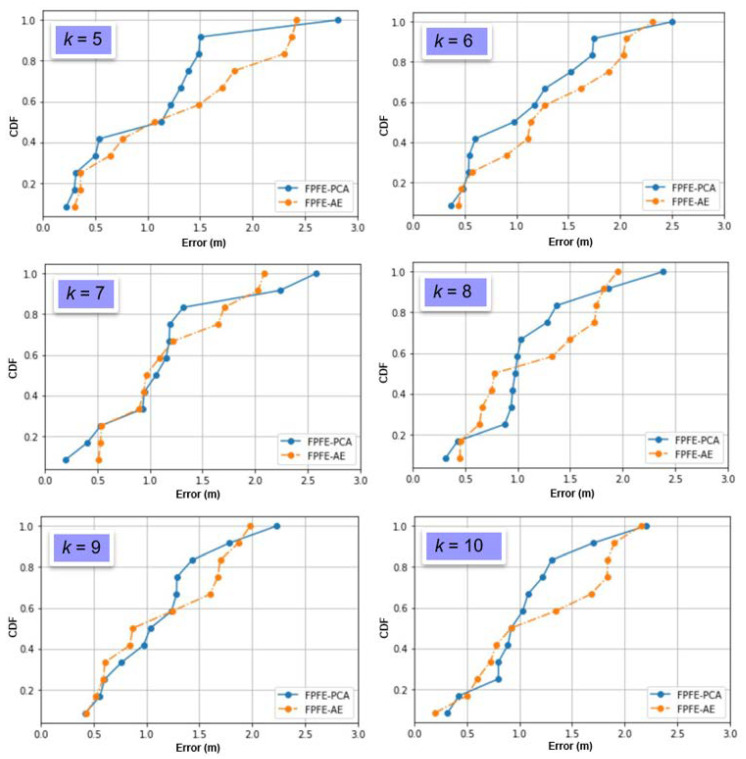
The localization error CDF curves of FPFE with 54 grid RPs for *k* RP candidates, where *k* = 5, 6, 7, 8, 9, and 10.

**Figure 11:**
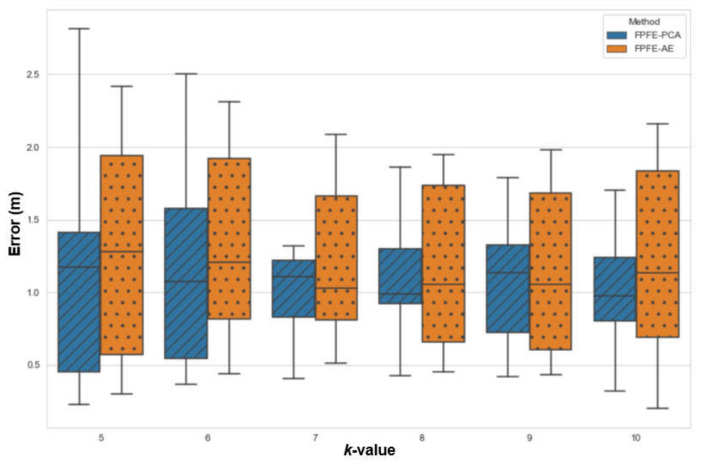
The localization error box and whisker plots of FPFE with 54 grid RPs for *k* RP candidates, where *k* = 5, 6, 7, 8, 9, and 10.

**Figure 12:**
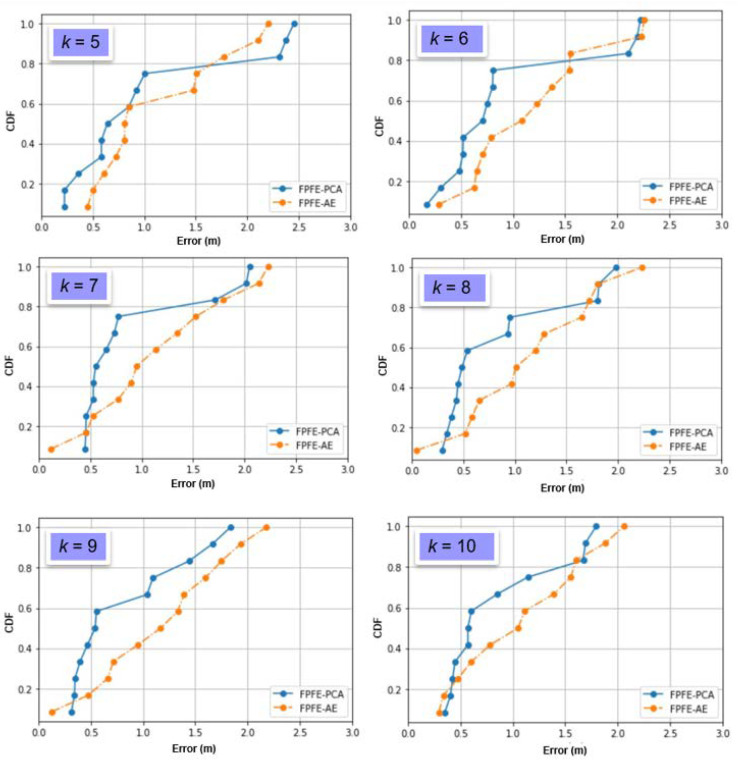
The localization error CDF curves of FPFE with 93 random RPs for *k* RP candidates, where *k* = 5, 6, 7, 8, 9, and 10.

**Figure 13:**
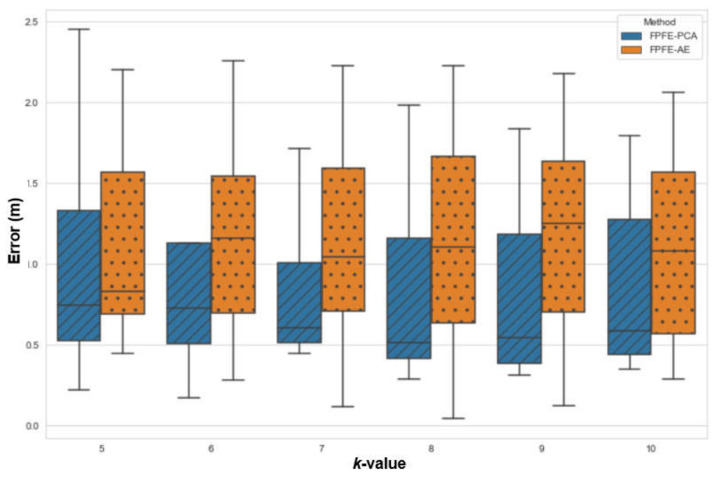
The localization error box and whisker plots of FPFE with 93 random RPs for *k* RP candidates, where *k* = 5, 6, 7, 8, 9, and 10.

**Figure 14:**
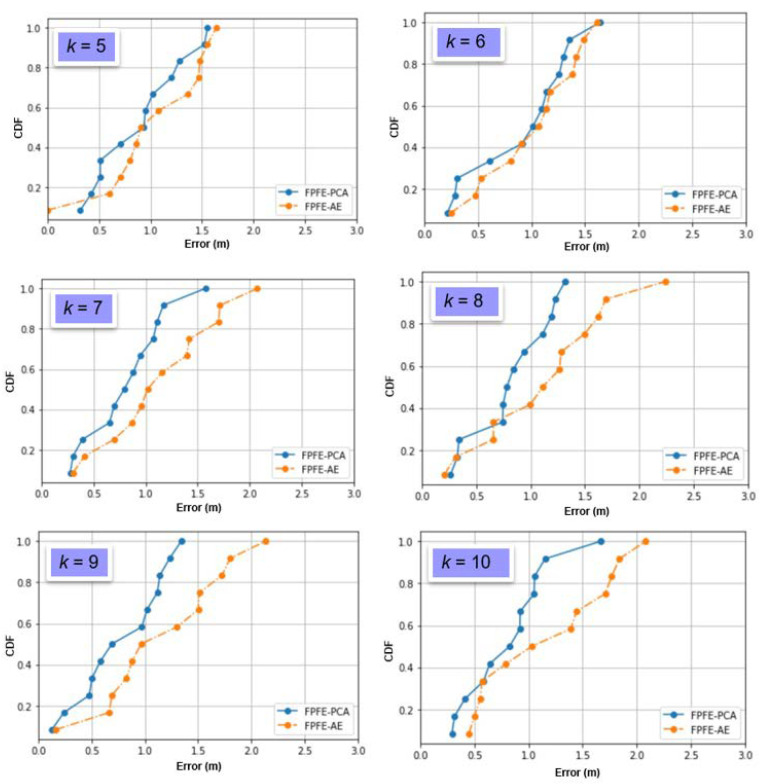
The localization error CDF curves of FPFE with 93 grid RPs for *k* RP candidates, where *k* = 5, 6, 7, 8, 9, and 10.

**Figure 15:**
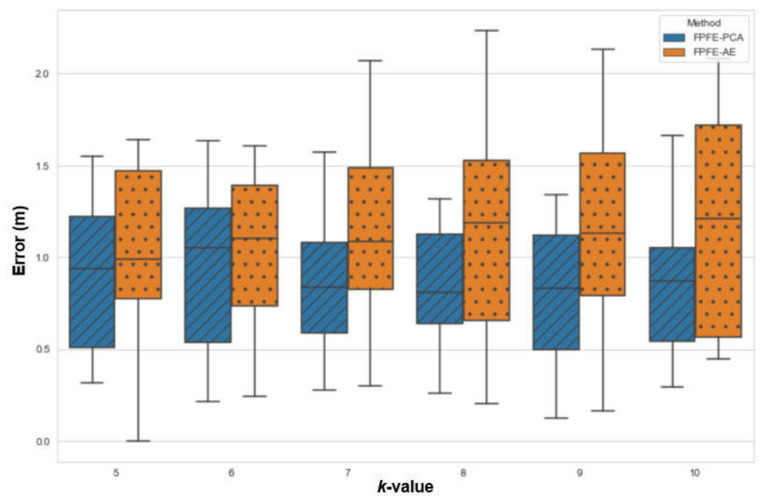
The localization error box and whisker plots of FPFE with 93 grid RPs for *k* RP candidates, where *k* = 5, 6, 7, 8, 9, and 10.

**Figure 16:**
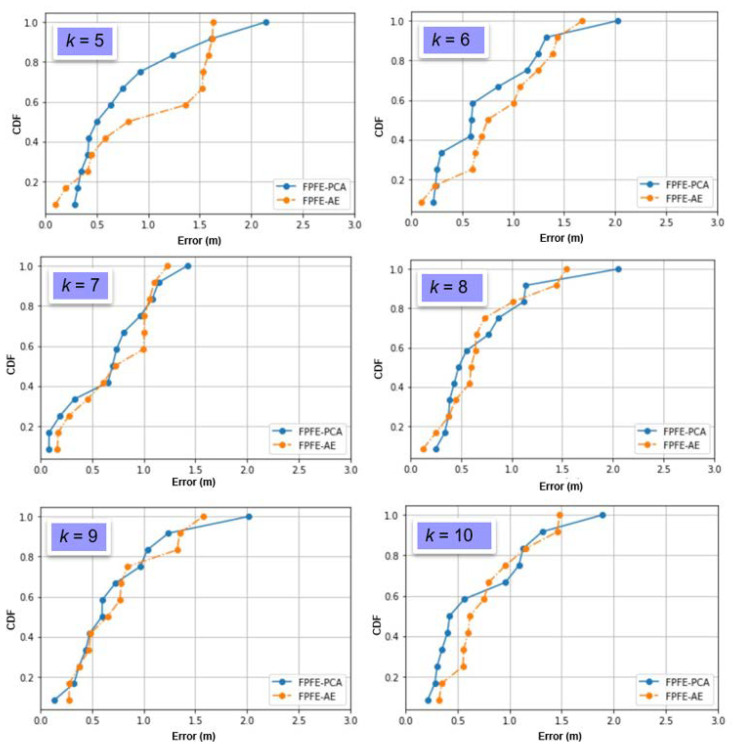
The localization error CDF curves of FPFE with 187 grid RPs for *k* RP candidates, where *k* = 5, 6, 7, 8, 9, and 10.

**Figure 17:**
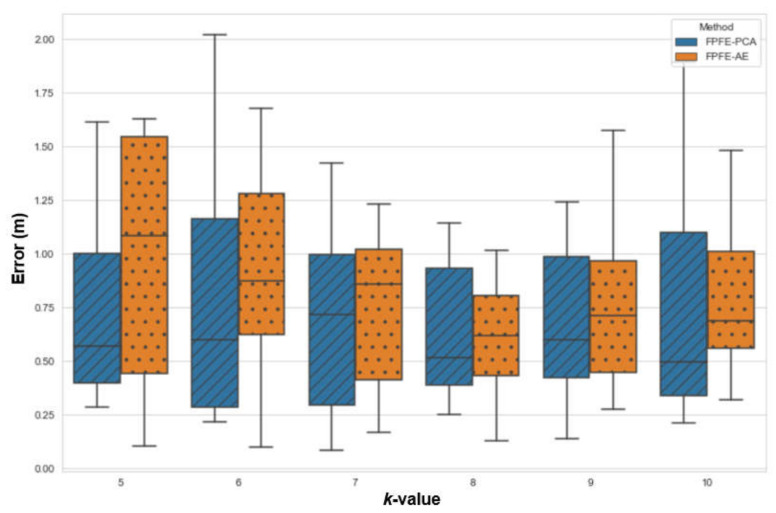
The localization error box and whisker plots of FPFE 187 grid RPs for *k* RP candidates, where *k* = 5, 6, 7, 8, 9, and 10.

The localization error of the FPFE method is evaluated for both the case using AE feature extraction, denoted as FPFE-AE, and the case using the PCA feature extraction denoted as FPFE-PCA. Figure 8, Figure 9, Figure 10, Figure 11, Figure 12, Figure 13, Figure 14, Figure 15, Figure 16 and Figure 17 show the localization errors of FPFE-AE and FPFE-PCA. Among the figures, Figure 8, Figure 10, Figure 12, Figure 14, and Figure 16 show the cumulative distribution function (CDF) curves for the localization errors of FPFE-AE and FPFE-PCA with *k* RP candidates, where *k* = 5, 6, 7, 8, 9, or 10. It can be observed from these figures that FPFE-AE and FPFE-PCA have similar CDF curves, but FPFE-PCA’s curves are usually on top of FPFE-AE’s. This means FPFE-PCA outperforms FPFE-AE. However, FPFE-PCA’s curves usually cover wider error ranges than FPFE-AE’s. This means FPFE-PCA has larger variances than FPFE-AE. This can also be observed from Figure 9, Figure 11, Figure 13, Figure 15, and Figure 17, which show the localization error box-whisker plots of FPFE-AE and FPFE-PCA for different *k* values, *k* = 5, 6, 7, 8, 9, or 10. In these box-whisker plots, the FPFE-PCA is usually taller (from the minimum to the maximum) and has larger boxes (from the first quartile *Q*_1_ to the third quartile *Q*_3_) than FPFE-AE. However, the FPFE-PCA usually has lower *Q*_1_ and *Q*_2_ (i.e., median) and usually has a lower *Q*_3_ than FPFE-AE.

**Figure 18:**
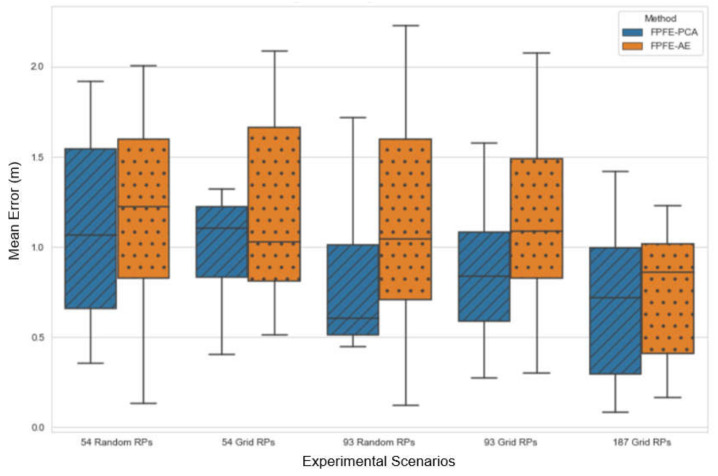
The localization error box and whisker plots of FPFE for different experimental scenarios with *k* = 7 RP candidates.

**Figure 19:**
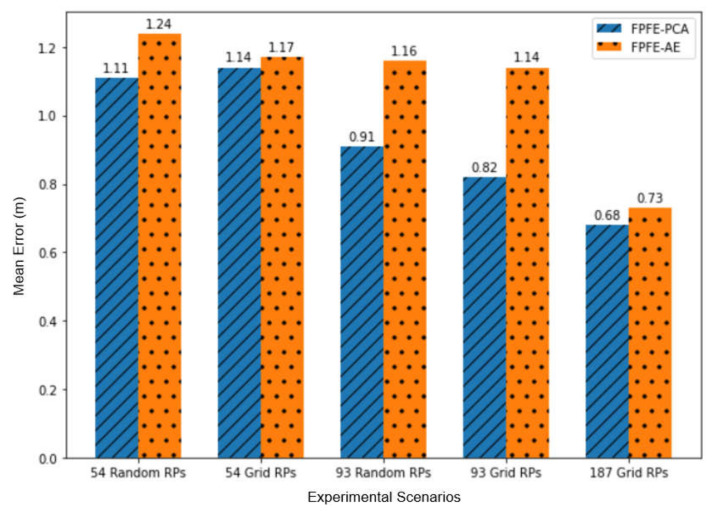
The mean localization errors of FPFE for different experimental scenarios with *k* = 7 RP candidates.

Figure 18 and Figure 19 show the localization error box-whisker plots and mean bar charts of FPFE for different experimental scenarios with *k* = 7 RP candidates. It can be observed from these two figures that FPFE-PCA is usually better than FPFE-AE under the same experimental scenario, i.e., the identical number of RPs and the identical RP setting (random or grid). It can also be observed that grid RPs result in better performance than random RPs and that more RPs lead to better performance. Furthermore, more RPs achieve better performance than fewer RPs. Or equivalently, smaller RP spacing leads to better performance than larger RP spacing. That is to say, the scenario of 189 grid RPs allows FPFE to achieve the best performance.

Table 1 shows the localization error statistics of FPFE-AE and FPFE-PCA under the scenario of 187 grid RPs for different *k* values, *k* = 5, 6, 7, 8, 9, or 10. The statistics include the maximum, median, mean, minimum, standard deviation, and variance. As shown in Table 1, FPFE-AE usually has smaller variances than PFPE-PCA. FPFE-AE has the smallest localization error of 0.09 m when *k* = 6, and it has the largest error of 1.67 m when *k* = 6. FPFE-PCA has the smallest error of 0.08 m when *k* = 7, and it has the largest error of 2.13 m when *k* = 5. Furthermore, FPFE-AE has the smallest mean (average error) of 0.70 m when *k* = 8, whereas FPFE-PCA has the smallest mean of 0.68 m when *k* = 7.

From the statistics in Table 1, as well as the CDF curves, box and whisker plots, and mean bar charts in Figure 8, Figure 9, Figure 10, Figure 11, Figure 12, Figure 13, Figure 14, Figure 15, Figure 16, Figure 17, Figure 18 and Figure 19, it can be observed that more RPs lead to better performance. Under the scenario of 187 grid RPS, FPFE-AE is more stable than FPFE-PCA, as FPFE-AE usually has smaller variances. Yet, PFE-PCA usually has better performance than FPFE-AE in terms of the localization error. This is because the PCA is based on the linear transformation that is effective when applied to a small dataset. On the contrary, the AE is based on the nonlinear transformation that can generate more effective results when applied to larger datasets. In experiments conducted in this research, the dataset fed into the PCA and AE is small, as it has only 800 or less data items. This may account for the better performance of FPFE-PCA. Furthermore, the AE model needs more time than PCA for model training (or model construction). In summary, FPFE-AE is recommended when stability is a major concern. However, FPFE-PCA is recommended to save computational resources and to be used in environments with small datasets.

Below, the Minkowski distance similarity measurements of fingerprint features are evaluated. As shown earlier, the Minkowski distance is also known as the L*p* norm distance. When *p* = 1, it is the Manhattan distance. When *p* = 2, it is the Euclidean distance. The evaluation is for *k* = 7 RP candidates under the scenario of 187 grid RPs, as the FPFE method has good performance for such a setting. Specifically, the evaluation is performed for the Manhattan distance, Euclidean distance, and Minkowski distance with *p* = *k* = 7 (i.e., the L_7_ norm distance). The evaluation results are presented in Table 2. It can be observed that the Minkowski distance with *p* = *k* = 7 (i.e., the L_7_ norm distance) makes both FPFE-AE and FPFE-PCA achieve the best performance.

### 4.3. Performance Comparison

The proposed FPFE method with *k* = 7 RPs is compared with five related BLE fingerprint-based localization methods [16,22,23,24,27]. The comparisons are for the cases of FPFE using AE feature extraction (FPFE-AE) and FPFE using the PCA feature extraction (FPFE-PCA). The methods are compared in the aspects of the localization area size, the number of BNs, and the minimum, the average, and the maximum localization errors. As shown in Section 2, Zuo et al. [16] used graph optimization to achieve the best result of 1.27 m on average. Martins et al. [22] utilized a Gaussian kernel-based fingerprinting concept to achieve errors that are less than 1.5 m for 90% of test cases. Subedi et al. [23] used a two-step fingerprint-based localization approach, resulting in an average localization error of 1.05 m. Li et al. [24] employed the eight-neighborhood template-matching mechanism to achieve an average localization error of 1.0 m. Dinh et al. [27] proposed a lightweight and reliable fingerprint-based method using PDR, bringing about the average and maximum localization errors of 0.81 and 2.11 m, respectively. Table 3 shows the comparisons of FPFE and other methods. By the comparison results, the proposed FPFE method achieves the average localization errors of 0.74 m (for FPFE-AE) and 0.68 m (for FPFE-PCA) and significantly outperforms the other related methods.

Since FPFE is based on the RF BLE technology, we compare, qualitatively, the BLE technology with other RF technologies, as well as with the optical, IR, and MS technologies, adopted by indoor localization methods. The comparison results in terms of the cost, coverage, and public infrastructure requirement are shown in Table 4. Using optical or vision-based TDs is medium cost since high-performance devices are needed to perform image processing for indoor localization. The drawback of optical technologies is the accuracy, and the coverage may be low because of the interference from numerous factors such as strong light, and motion blur. IR devices have the LOS limitation, which makes the coverage low, whereas the cost of IR devices is medium. MSs have low costs, whereas their coverage is medium even though errors are accumulated over a distance. Wi-Fi access points have medium costs, whereas they have medium coverage. The UWB technology has a high setup cost, whereas its coverage is low since it is for short-range communications. BLE devices have low cost, and their coverage is low as it is also for short-range communications. Cellular technology has high coverage and has a high cost because it needs to set up the costly public infrastructure of extensive base stations using licensed frequency bands. Unlike cellular technology, other technologies need no pre-established public infrastructure.

### 4.4. An FPFE Application

**Figure 20:**
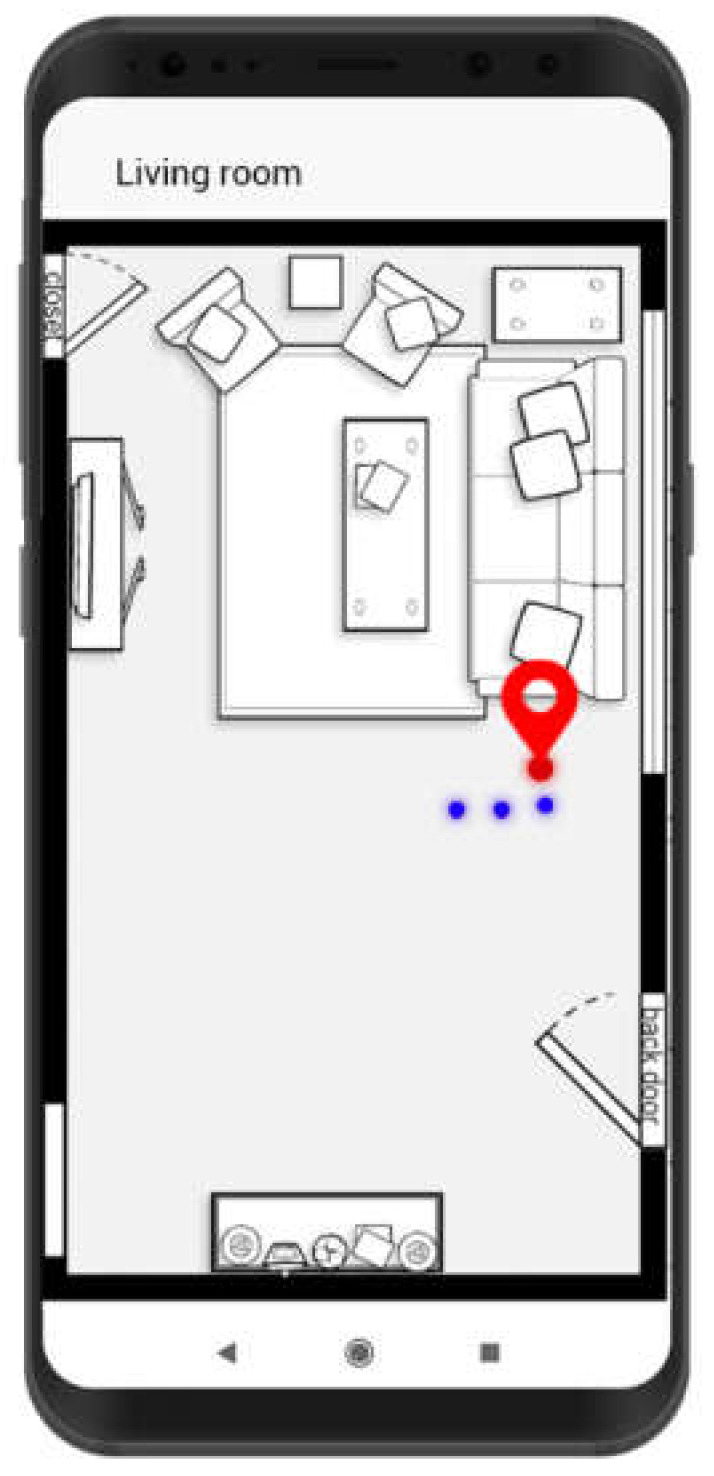
Screenshot of a smart home app prototype using FPFE.

This subsection describes a prototype application based on an FPFE for smart homes. The application is under development and intended to be implemented in the form of an Android app for assisting people to live smartly at home. A screenshot of the app is shown in Figure 20. In the screenshot, the red indicator on the right side is the current user location, and the blue dots are several latest locations of the user.

With the aid of FPFE, the app offers convenient services to residents staying at home. For example, FPFE can enable the app to make a home more energy-efficient and more context-aware. When a user moves to a spot in the living room, the lamp near the spot will be turned on. The lamp will be automatically turned off when the user moves far away from the spot or leaves the living room for a long enough time. For another example, FPFE can enable the app to remind users if they forget to lock the door on leaving home. The app can even lock the door on the request of users for the sake of security.

## 5. Conclusions

This paper proposes FPFE, a BLE fingerprint-based indoor localization method on the basis of fingerprint feature extraction using either AE or PCA. FPFE also relies on the Minkowski distance for measuring the similarity between the features of the TD and all RPs to select *k* RP candidates for TD location estimation. FPFE is compared with other BLE fingerprint-based methods [16,22,23,24,27] to show its superiority in terms of the location error. The compared methods are the fingerprint-based and range-based graph optimization methods [16], the fingerprint-based Gaussian kernel method [22], the fingerprint-based weighted centroid method [23], the fingerprint-based eight-neighborhood template matching method [24], and the fingerprint-based PDR method [27]. Consequently, FPFE achieves an average localization error of 0.7 m with AE feature extraction for *k* = 8, and an error of 0.68 m with PCA feature extraction for *k* = 7. An Android app for smart homes utilizing FPFE is under development. The accurate localization results of FPFE enable the app to make living at home more energy-efficient and more context-aware.

Tiglao et al. [40] reviewed the state-of-the-art of smartphone-based indoor localization. The authors mentioned ten open challenges in indoor localization: 3D localization, hardware dependency, power consumption, accuracy, heading inference, heterogeneous hardware, latency improvement, step counting, map generation, and multi-floor localization. Our research contributes to improving the accuracy with low power consumption to fill the gap between FPFE and other BLE indoor localization methods.

However, the proposed FPFE method still has room for improvement. For example, FPFE takes about 2 min to collect fingerprint data for an RP, and a total of 6.2 h for 187 RPs. Collecting RP’s fingerprint data is a time-consuming and labor-intensive task. In the future, we plan to apply the ray tracing (RT) fingerprint estimation mechanism [41] to perform the task to save time and labor. RT fingerprint estimation usually takes LOS, specular reflection and diffraction, and diffusion scattering into consideration. However, we need to handle significant discrepancies between measured fingerprints and RT-estimated fingerprints in the situation of non-line-of-sight (NLOS) and high scattering complexity.

When the environment changes (e.g., the temperature varies or the TD alters), the performance of FPFE degrades. For example, when the TD alters from an Asus phone to a Sony phone, the mean localization errors become 2.07 m for FPFE-AE and 2.03 m for FPFE-PCA. It is thus necessary to recollect fingerprint data and retrain PCA and AE models. Using a desktop computer with a 3.4 GHz Core i5-7500 CPU and 16 GB RAM to construct (or train) a PCA (resp., AE) model for extracting features of 187 RPs takes around 2 s (resp., 7 min). The model training time should be reduced so that FPFE can react to changes in the environment in less time. In the future, we plan to employ the transfer learning concept [42] to train models for a target domain (i.e., a new environment) by leveraging the models for the source domain (i.e., the old environment) with only a few training data. By deep learning, we only need to collect fingerprint data of a few PRs for training AE or PCA models. In this way, the time consumed in collecting fingerprint data and training models can be significantly reduced.

FPFE is currently applied to a 5 m × 8 m indoor area for the purpose of localization. The localization area should be enlarged so that FPFE can be applied to applications with large localization areas. In the future, we plan to apply the FPFE method to large indoor areas. Furthermore, we also plan to extend FPFE to continuously track the locations of the TD that arbitrarily moves in a large indoor area with advanced technology, like Bayesian inference and long short-term memory (LSTM) neural networks.

FPFE relies on BLE BNs to perform indoor localization. Since BLE BNs are powered by batteries, they may sometimes fail, degrading FPFE performance and even preventing FPFE from working. Therefore, we need to deal with BN faults to make FPFE fault-tolerant. Carvalho et al. [10] considered two types of failures, momentary failures and permanent failures, and designed a fault-tolerant indoor localization system through different recurrent neural networks (RNNs) such as simple RNN, gated recurrent unit (GRU) neural network, and the long short-term memory (LSTM) neural network. In the future, we plan to utilize novel neural networks such as the long short-term cognitive network (LSTCN) [43] to make FPFE fault-tolerant.

The localization accuracy of FPFE using the BLE technology is sub-meter, which can meet the requirements of some applications such as smart homes. In light of the research result of reference [44], we plan to study the accuracy bound of FPFE and will try to push it to the limit of the bound. Alternatively, we may need to use different RF technologies to develop novel localization methods for specific location-based applications that require centimeter or even sub-centimeter accuracy. In the future, we plan to investigate fingerprint-based methods [45,46,47,48,49,50,51,52,53,54,55] using different RF technologies, as described below.

Many fingerprint-based localization methods rely on RF fingerprints to achieve the submeter, centimeter, or even sub-centimeter level of localization accuracy. Those methods use different RF technologies, including the Wi-Fi frequency-hopping approach [45], UWB spatial signal prediction [46], IEEE 802.11ad mmWaves [47], 5G massive MIMO [48,49], cellular time-reversal technique [50], Wi-Fi channel responses from multiple OFDM subcarriers [51], Wi-Fi time-reversal radio transmission [52], Wi-Fi ray tracing [53], BLE ray tracing [41], and 6G reconfigurable intelligent surface (RISs) [54,55]. Three types of diversities are adopted by the methods to ink fingerprints, which are spatial diversity [47,48,49], spectral diversity [45,46,50,51,52], and configurational diversity [54,55]. Methods based on different diversity to ink fingerprints need different hardware support and spectrum allotment, causing various advantages and disadvantages. We plan to investigate the advantages and disadvantages to find appropriate diversities for designing indoor localization methods achieving desirable localization accuracy with affordable resources consumed. We have also noticed that the search [56] proposes using wave fingerprints (WFPs) for localization in dynamic complex environments. It investigates the correlation of WFRs and shows that WFP localization is possible even in a highly perturbed environment. Using WFPs for indoor localization is thus a promising research direction.

## Figures and Tables

**Table 1:** FPFE localization error statistics.

Methods	Stats.	*k* = 5	*k* = 6	*k* = 7	*k* = 8	*k* = 9	*k* = 10
FPFE-AE	Max	1.62	1.67	1.23	1.54	1.57	1.48
	Median	1.08	0.87	0.85	0.61	0.71	0.68
	Mean	0.98	0.90	0.73	0.70	0.76	0.80
	Min	0.13	0.09	0.16	0.12	0.27	0.32
	Std	0.58	0.46	0.37	0.41	0.42	0.37
	Var	0.34	0.21	0.14	0.17	0.18	0.14
FPFE-PCA	Max	2.13	2.02	1.43	2.05	2.01	1.8
	Median	0.56	0.59	0.71	0.51	0.59	0.49
	Mean	0.79	0.77	0.68	0.73	0.74	0.74
	Min	0.28	0.21	0.08	0.25	0.13	0.21
	Std	0.56	0.53	0.41	0.49	0.49	0.50
	Var	0.31	0.28	0.17	0.24	0.24	0.25

**Table 2:** Evaluations for different distance similarity measurements for *k* = 7 RP candidates under the scenario of 187 grid RPs.

Methods	Manhattan Distance (m)	Euclidean Distance (m)	Minkowski Distance (m)
FPFE-AE	0.97	0.91	0.73
FPFE-PCA	0.91	0.80	0.68

**Table 3:** Comparisons of FPFE-AE and FPFE-PCA with other fingerprint-based methods.

Research	Method	Area Size (m)	Number of BNs	Minimum Error (m)	Average Error (m)	Maximum Error (m)
Zuo et al. [16]	Fingerprint-based and range-based graph optimization	90 × 37	24		1.27	3.07
Martins et al. [22]	Fingerprint-based Gaussian kernel	200 × 40	45	-	1.5	-
Subedi et al. [23]	Fingerprint-based weighted centroid	93.3 × 2.67	28	-	1.05	-
Li et al. [24]	Fingerprint-based eight-neighborhood template matching	8 × 8	4	-	1.0	-
Dinh et al. [27]	Fingerprint-based PDR	15 × 25	8	-	0.81	2.114
This Research	FPFE-AE	5 × 8	4	0.16	0.73	1.23
This Research	FPFE-PCA	5 × 8	4	0.08	0.68	1.43

**Table 4:** Comparisons of RF technologies for indoor localization.

Devices	Cost	Coverage	Public Infrastructure
Optical	Medium	Low	No
Infrared	Medium	Low	No
Mechanical sensor	Low	Medium	No
BLE	Low	Low	No
Wi-Fi	Medium	Medium	No
UWB	High	Low	No
Cellular	High	High	Yes

## References

[1] Zafari F., Gkelias A., Leung K.K. (2019). A Survey of Indoor Localization Systems and Technologies. IEEE Commun. Surv. Tutor..

[2] Bazo R., da Costa C.A., Seewald L.A., da Silveira L.G., Antunes R.S., Righi R.D.R., Rodrigues V.F. (2021). A Survey About Real-Time Location Systems in Healthcare Environments. J. Med. Syst..

[3] Witrisal K., Meissner P., Leitinger E., Shen Y., Gustafson C., Tufvesson F., Haneda K., Dardari D., Molisch A.F., Conti A. (2016). High-Accuracy Localization for Assisted Living: 5G systems will turn multipath channels from foe to friend. IEEE Signal Process. Mag..

[4] Rácz-Szabó A., Ruppert T., Bántay L., Löcklin A., Jakab L., Abonyi J. (2020). Real-Time Locating System in Production Management. Sensors.

[5] Sakpere W., Oshin M.A., Mlitwa N.B. (2017). A State-of-the-Art Survey of Indoor Positioning and Navigation Systems and Technologies. S. Afr. Comput. J..

[6] Geok T.K., Aung K.Z., Aung M.S., Soe M.T., Abdaziz A., Liew C.P., Hossain F., Tso C.P., Yong W.H. (2020). Review of Indoor Positioning: Radio Wave Technology. Appl. Sci..

[7] Want R., Hopper A., Falcão V., Gibbons J. (1992). The active badge location system. ACM Trans. Inf. Syst..

[8] Arbula D., Ljubic S. (2020). Indoor Localization Based on Infrared Angle of Arrival Sensor Network. Sensors.

[9] Subbu K.P., Gozick B., Dantu R. (2013). Locate Me: Magnetic-fields-based indoor localization using smartphones. ACM Trans. Intell. Syst. Technol..

[10] Carvalho E.C., Ferreira B.V., Filho G.P.R., Gomes P.H., Freitas G.M., Vargas P.A., Ueyama J., Pessin G. Towards a Smart Fault Tolerant Indoor Localization System Through Recurrent Neural Networks. Proceedings of the International Joint Conference on Neural Networks (IJCNN 2019).

[11] Dian F.J., Yousefi A., Lim S. A practical study on Bluetooth Low Energy (BLE) throughput. Proceedings of the IEEE 9th Annual Information Technology, Electronics and Mobile Communication Conference (IEMCON 2018).

[12] Tiemann J., Wietfeld C. (2019). Scalability, Real-Time Capabilities, and Energy Efficiency in Ultra-Wideband Localization. IEEE Trans. Ind. Inform..

[13] Ma Y., Zhou G., Wang S. (2019). WiFi sensing with channel state information: A survey. ACM Comput. Surv..

[14] Huang S., Zhao K., Zheng Z., Ji W., Li T., Liao X. (2021). An Optimized Fingerprinting-Based Indoor Positioning with Kalman Filter and Universal Kriging for 5G Internet of Things. Wirel. Commun. Mob. Comput..

[15] Jiang J.-R., Subakti H., Chen C.-C., Sakai K. (2019). PINUS: Indoor Weighted Centroid Localization with Crowdsourced Calibration. Communications in Computer and Information Science.

[16] Zuo Z., Liu L., Zhang L., Fang Y. (2018). Indoor Positioning Based on Bluetooth Low-Energy Beacons Adopting Graph Optimization. Sensors.

[17] Li G., Geng E., Ye Z., Xu Y., Lin J., Pang Y. (2018). Indoor Positioning Algorithm Based on the Improved RSSI Distance Model. Sensors.

[18] Giovanelli D., Farella E., Fontanelli D., Macii D. Bluetooth-Based Indoor Positioning Through ToF and RSSI Data Fusion. Proceedings of the International Conference on Indoor Positioning and Indoor Navigation (IPIN 2018).

[19] Tegou T., Kalamaras I., Votis K., Tzovaras D. A low-cost room-level indoor localization system with easy setup for medical applications. Proceedings of the 11th IFIP Wireless and Mobile Networking Conference (WMNC 2018).

[20] Mussina A., Aubakirov S. RSSI Based Bluetooth Low Energy Indoor Positioning. Proceedings of the IEEE 12th International Conference on Application of Information and Communication Technologies (AICT 2018).

[21] Mackey A., Spachos P., Plataniotis K. Enhanced Indoor Navigation System with Beacons and Kalman Filters. Proceedings of the IEEE Global Conference on Signal and Information Processing (GlobalSIP 2018).

[22] Martins P., Abbasi M., Sá F., Celiclio J., Morgado F., Caldeira F. (2019). Intelligent beacon location and fingerprinting. Procedia Comput. Sci..

[23] Subedi S., Gang H.-S., Ko N.Y., Hwang S.-S., Pyun J.-Y. (2019). Improving Indoor Fingerprinting Positioning With Affinity Propagation Clustering and Weighted Centroid Fingerprint. IEEE Access.

[24] Li M., Zhao L., Tan D., Tong X. (2019). BLE Fingerprint Indoor Localization Algorithm Based on Eight-Neighborhood Template Matching. Sensors.

[25] Malekzadeh P., Mohammadi A., Barbulescu M., Plataniotis K. (2019). STUPEFY: Set-Valued Box Particle Filtering for Bluetooth Low Energy-Based Indoor Localization. IEEE Signal Process. Lett..

[26] Mouhammad C.S., Allam A., Abdel-Raouf M., Shenouda E., Elsabrouty M. BLE Indoor Localization based on Improved RSSI and Trilateration. Proceedings of the 7th International Japan-Africa Conference on Electronics, Communications, and Computations, (JAC-ECC 2019).

[27] Dinh T.-M.T., Duong N.-S., Sandrasegaran K. (2020). Smartphone-Based Indoor Positioning Using BLE iBeacon and Reliable Lightweight Fingerprint Map. IEEE Sens. J..

[28] Kluge T., Groba C., Springer T. Trilateration, Fingerprinting, and Centroid: Taking Indoor Positioning with Bluetooth LE to the Wild. Proceedings of the IEEE 21st International Symposium on “A World of Wireless, Mobile and Multimedia Networks” (WoWMoM 2020).

[29] Li Z., Cao J., Liu X., Zhang J., Hu H., Yao D. A Self-Adaptive Bluetooth Indoor Localization System using LSTM-based Distance Estimator. Proceedings of the 29th International Conference on Computer Communications and Networks (ICCCN 2020).

[30] Pakanon N., Chamchoy M., Supanakoon P. Study on Accuracy of Trilateration Method for Indoor Positioning with BLE Beacons. Proceedings of the 6th International Conference on Engineering, Applied Sciences and Technology (ICEAST 2020).

[31] Kotrotsios K., Orphanoudakis T. Accurate Gridless Indoor Localization Based on Multiple Bluetooth Beacons and Machine Learning. Proceedings of the 7th International Conference on Automation, Robotics and Applications (ICARA 2021).

[32] Zhu Y., Luo X., Guan S., Wang Z. Indoor Positioning Method Based on WiFi/Bluetooth and PDR Fusion Positioning. Proceedings of the 13th International Conference on Advanced Computational Intelligence (ICACI 2021).

[33] Hu Q., Wu F., Wong R.K., Millham R.C., Fiaidhi J. (2021). A novel indoor localization system using machine learning based on bluetooth low energy with cloud computing. Computing.

[34] Nessa A., Adhikari B., Hussain F., Fernando X.N. (2020). A Survey of Machine Learning for Indoor Positioning. IEEE Access.

[35] Kunang Y.N., Nurmaini S., Stiawan D., Zarkasi A., Firdaus, Jasmir F. Automatic Features Extraction Using Autoencoder in Intrusion Detection System. Proceedings of the International Conference on Electrical Engineering and Computer Science (ICECOS 2018).

[36] Wang Z., Zhang X., Wang W., Shi L., Huang C., Wang J., Zhang Y. Deep Convolutional Auto-Encoder based Indoor Light Positioning Using RSS Temporal Image. Proceedings of the IEEE International Symposium on Broadband Multimedia Systems and Broadcasting (BMSB 2019).

[37] Zhang L., Tan T., Gong Y., Yang W. (2019). Fingerprint Database Reconstruction Based on Robust PCA for Indoor Localization. Sensors.

[38] Khaldi B., Harrou F., Cherif F., Sun Y. Improving robots swarm aggregation performance through the Minkowski distance function. Proceedings of the 6th International Conference on Mechatronics and Robotics Engineering (ICMRE 2020).

[39] Subakti H., Liang H.-S., Jiang J.-R. Indoor Localization with Fingerprint Feature Extraction. Proceedings of the IEEE Eurasia Conference on IOT, Communication and Engineering (ECICE 2020).

[40] Tiglao N.M., Alipio M., Cruz R.D., Bokhari F., Rauf S., Khan S.A. (2021). Smartphone-based indoor localization techniques: State-of-the-art and classification. Measurement.

[41] Renaudin O., Zemen T., Burgess T. Ray-Tracing Based Fingerprinting for Indoor Localization. Proceedings of the IEEE 19th International Workshop on Signal Processing Advances in Wireless Communications (SPAWC 2018).

[42] Agarwal N., Sondhi A., Chopra K., Singh G. (2020). Transfer Learning: Survey and Classification. Advances in Intelligent Systems and Computing.

[43] Nápoles G., Grau I., Jastrzebska A., Salgueiro Y. (2021). Long Short-term Cognitive Networks. arXiv.

[44] del Hougne M., Gigan S., del Hougne P. (2021). Deeply Subwavelength Localization with Reverberation-Coded Aperture. Phys. Rev. Lett..

[45] Chen C., Chen Y., Han Y., Lai H.-Q., Liu K.J.R. (2016). Achieving Centimeter Accuracy Indoor Localization on WiFi Platforms: A Frequency Hopping Approach. IEEE Internet Things J..

[46] Steiner C., Wittneben A. (2011). Efficient Training Phase for Ultrawideband-Based Location Fingerprinting Systems. IEEE Trans. Signal Process..

[47] Vari M., Cassioli D. mmWaves RSSI indoor network localization. Proceedings of the IEEE International Conference on Communications Workshops (ICC 2014).

[48] Savic V., Larsson E.G. Fingerprinting-Based Positioning in Distributed Massive MIMO Systems. Proceedings of the IEEE 82nd Vehicular Technology Conference (VTC2015-Fall).

[49] Vieira J., Leitinger E., Sarajlic M., Li X., Tufvesson F. Deep convolutional neural networks for massive MIMO fingerprint-based positioning. Proceedings of the IEEE 28th Annual International Symposium on Personal, Indoor, and Mobile Radio Communications (PIMRC 2017).

[50] Jin Y., O’Donoughue N., Moura J.M.F. Position location by time reversal in communication networks. Proceedings of the 2008 IEEE International Conference on Acoustics, Speech and Signal Processing.

[51] Sen S., Radunovic B., Choudhury R.R., Minka T. You are facing the Mona Lisa: Spot localization using PHY layer information. Proceedings of the 10th International Conference on Mobile Systems, Applications, and Services.

[52] Wu Z.-H., Han Y., Chen Y., Liu K.J.R. (2015). A Time-Reversal Paradigm for Indoor Positioning System. IEEE Trans. Veh. Technol..

[53] Del Corte-Valiente A., Gómez-Pulido J.M., Gutiérrez-Blanco O., Castillo-Sequera J.L. (2019). Localization Approach Based on Ray-Tracing Simulations and Fingerprinting Techniques for Indoor–Outdoor Scenarios. Energies.

[54] Alexandropoulos G.C., Shlezinger N., del Hougne P. (2021). Reconfigurable Intelligent Surfaces for Rich Scattering Wireless Communications: Recent Experiments, Challenges, and Opportunities. IEEE Commun. Mag..

[55] Abu-Shaban Z., Keykhosravi K., Keskin M.F., Alexandropoulos G.C., Seco-Granados G., Wymeersch H. (2020). Near-field localization with a reconfigurable intelligent surface acting as lens. arXiv.

[56] Del Hougne P. (2020). Robust position sensing with wave fingerprints in dynamic complex propagation environments. Phys. Rev. Res..

